# NGMA-2. Dual sgRNA-directed PD-L1 knockout in human glioblastoma cells using the CRISPR/Cas9 system

**DOI:** 10.1093/noajnl/vdab070.017

**Published:** 2021-07-05

**Authors:** Javier Fierro, Jake Dipasquale, Rocio Aguilar, Joshua Perez, An Tran, Chris Factoriza, Huanyu Dou

**Affiliations:** Texas Tech University Health Sciences Center El Paso, El Paso, TX, USA

## Abstract

Glioblastoma multiforme (GBM) is an astrocyte derived brain tumor. It induces an immunosuppressive microenvironment by exploiting immune checkpoints such as the PD-1/PD-L1 pathway. Targeting the PD-1/PD-L1 pathway for immunotherapy is a promising new avenue for treating GBM, but more work is needed to develop a safe and effective method for clinical applications. We identified two sgRNA sequences located on PD-L1 exon 3. The first sgRNA recognized the forward strand of human PD-L1 near the beginning of exon 3 and cuts at approximately base pair 82 (g82). The second sgRNA recognized the reverse strand of exon 3 and cuts at base pair 165 (g165). Two sgRNAs, g82 and g165, created an 83bp deletion in the genomic sequence that can lead to the production of a non-functional PD-L1 protein. A homology-directed repair template (HDR) containing an in-frame stop codon was designed to enhance PD-L1 knockout specificity and efficiency. Both g82 and g165 were cloned into the CRISPR/Cas9 plasmid, and was co-transfected with the added HDR template. T7E1, qRT-PCR and western blot analysis determined that the dual sgRNA CRISPR/Ca9 system knocked out both endogenous (80%) and exogenous (64%) PD-L1 in U87 cells and PD-L1 overexpression U87 cells, respectively. Deletion of PD-L1 reduced U87 migration and proliferation, while PD-L1 overexpression promoted tumor growth and tumor-associated macrophage polarization. Together, deletion of both membrane and cytoplasmic PD-L1 altered the PD-L1-associated immunosuppressive environment and prevented tumor progression and migration. Thus, a dual sgRNA CRISPR/Cas9 gene-editing system is a promising avenue for anti-GBM immunotherapy.

